# Pharmacogenetic Predictors of Metformin Response in Metabolic Syndrome and Type 2 Diabetes: Evidence from a Cohort Study in Kazakhstan

**DOI:** 10.1155/jdr/1568889

**Published:** 2025-09-26

**Authors:** Altynay T. Imangaliyeva, Nurgul S. Sikhayeva, Serik A. Baidurin, Bibigul A. Suleimenova, Aliya A. Romanova, Elena V. Zholdybayeva, Serik A. Shaimerdenov, Aigul Sh. Kuanysheva, Aidos Bolatov

**Affiliations:** ^1^Department of Internal Medicine With Courses in Nephrology, Hematology, Allergology and Immunology, Astana Medical University, Astana, Kazakhstan; ^2^National Center for Biotechnology, National Holding “Qazbiopharm”, Astana, Kazakhstan; ^3^Administration Unit, “Salauatty Astana” LLC, Astana, Kazakhstan; ^4^Administration Unit, City Polyclinic №4, Astana, Kazakhstan; ^5^Administration Unit, City Polyclinic №7, Astana, Kazakhstan; ^6^Shenzhen University Medical School, Shenzhen University, Shenzhen, Guangdong, China; ^7^Division of Strategic Development and Science, “Human Research & Development” LLP, Astana, Kazakhstan

**Keywords:** Kazakhstan, metabolic syndrome, metformin, pharmacogenetics, precision medicine, Type 2 diabetes mellitus

## Abstract

**Background:** Type 2 diabetes mellitus (T2DM) and metabolic syndrome (MetS) are highly prevalent in Kazakhstan, with metformin as the first-line therapy. However, individual variability in treatment response and adverse effects necessitates pharmacogenetic evaluation. This study is aimed at assessing the clinical and biochemical efficacy of metformin in Kazakh patients with MetS, T2DM, or both and at identifying pharmacogenetic markers associated with treatment response and tolerability.

**Methods:** A prospective cohort of 206 Kazakh patients (MetS: *n* = 44, MetS + T2DM: *n* = 123, T2DM: *n* = 39) was enrolled. Clinical, anthropometric, and biochemical parameters were measured at baseline and 3 months after metformin therapy. Ten SNPs previously implicated in metformin pharmacogenetics were genotyped. Associations with treatment efficacy (HbA1c reduction and glycemic goal achievement) and dyspeptic side effects were analyzed using ANOVA, logistic regression, and chi-square tests.

**Results:** Metformin significantly improved weight, blood pressure, glucose, and lipid profiles across all groups. The SNPs rs2289669 (SLC47A1) and rs3792269 (CAPN10) were significantly associated with greater HbA1c reduction, glycemic goal achievement, and risk of dyspepsia. The rs11212617 variant showed a modest association with treatment efficacy.

**Conclusions:** Metformin is effective across diverse metabolic phenotypes in the Kazakh population, with genetic variation contributing to interindividual differences in efficacy and tolerability. Pharmacogenetic profiling may improve personalized diabetes care.

## 1. Introduction

Type 2 diabetes mellitus (T2DM) is a rapidly growing global health issue, characterized by insulin resistance and relative insulin deficiency, which leads to complications such as cardiovascular disease, kidney failure, and neuropathy [1]. In 2021, over 529 million people were living with diabetes, with a global prevalence of 6.1% [2]. The increasing burden of T2DM is largely driven by rising obesity rates linked to urbanization and lifestyle changes, especially in low- and middle-income countries [3, 4]. These regions have experienced the highest increases in diabetes-related incidence, mortality, and disability. T2DM is associated with a 15% higher risk of early death and up to 20 years of reduced life expectancy [4].

Metabolic syndrome (MetS), a condition involving abdominal obesity, high blood pressure, elevated glucose, high triglycerides, and low HDL cholesterol, is a major risk factor for T2DM, cardiovascular disease, and stroke [5–7]. Its prevalence ranges from 27.4% to 39.0% [8], and it affects 70%–80% of individuals with T2DM [5]. Given the growing burden of T2DM and MetS, particularly in developing regions, improving early detection and management strategies is essential [4].

Kazakhstan, a Central Asian country and former Soviet republic, has experienced a rapid rise in the burden of T2DM and MetS over recent decades. Between 2014 and 2019, the prevalence of T2DM nearly doubled from 1208 to 2075 cases per 100,000 population, while related mortality increased sixfold [9, 10]. A systematic review reported that the recorded prevalence quadrupled between 2004 and 2021 (from 0.8% to 3.74%) [11]. Survey-based estimates suggest an even higher actual burden, around 8%, according to both the World Health Organization (WHO) STEPS and NOMAD studies, with over half of cases undiagnosed [10, 12]. Prevalence increases with age and urban residence, affecting 16.3% of urban adults aged 50–75, compared to 8.6% in rural areas [13]. Male sex, older age, and Kazakh ethnicity are associated with increased T2DM-related mortality [9]. Despite the implementation of a National Diabetes Screening Program in 2011, many individuals remain at elevated risk due to widespread metabolic risk factors. About 54% of the population is overweight or obese, and 45% has dyslipidemia [14]. MetS, a major contributor to T2DM and cardiovascular disease, is also prevalent: National estimates range from 14.7% in the general population to over 38%–45% in older adults [15] and reach 21.8%–25.6% in regional studies depending on the criteria used [16]. Taken together, these estimates place Kazakhstan within the European average of 20%–30% MetS prevalence [17], with some sources estimating national rates as high as 40% [18]. Collectively, these trends underscore the urgent need for improved screening, early detection, and personalized management strategies.

Metformin is a widely prescribed first-line medication for the treatment of T2DM due to its proven ability to improve insulin sensitivity, reduce hyperglycemia, and lower cardiometabolic risk. Its therapeutic benefits extend beyond glycemic control, positively influencing multiple components of MetS, including dysglycemia, obesity, and liver dysfunction [19]. Although there is currently no standardized treatment for MetS as a distinct clinical entity, metformin addresses many of its core features such as insulin resistance, dyslipidemia, and hypertension, making it a potentially valuable agent in preventing progression to T2DM and cardiovascular disease [20]. Emerging evidence also supports the use of metformin in individuals without overt carbohydrate metabolism disorders. In populations with unhealthy lifestyle patterns, metformin has shown potential to mitigate the harmful effects of a high-fat diet even before the onset of obesity or prediabetes [21]. Furthermore, studies indicate that metformin may be associated with improved survival among individuals with T2DM and MetS [22]. Metabolomic analyses have provided additional insight into metformin's systemic impact, showing that its use restores several dysregulated metabolites in obese diabetic patients to normal levels, particularly those involved in amino acid metabolism and the urea cycle [23]. These findings underscore metformin's expanding therapeutic value in managing early metabolic dysfunction and its potential for broader clinical application.

Metformin remains the most widely prescribed first-line therapy for T2DM, yet its glycemic effectiveness varies significantly among individuals. This variability is influenced by clinical and genetic factors, particularly polymorphisms in genes involved in metformin absorption, transport, and elimination—most notably SLC22A1, SLC22A2, and SLC22A3 [24]. Several single nucleotide polymorphisms (SNPs) in these genes have been associated with differential glycemic responses: For example, SLC22A3 rs12194182 was linked to lower glycated hemoglobin (HbA1c) in Jordanian patients [25], while SLC22A1 rs12208357 and rs594709, and SLC22A3 rs2076828, have also been implicated in reduced or improved response [26–28]. Beyond these transporters, variants such as SLC47A1 rs2289669 and SLC47A2 rs12943590 have been associated with enhanced metformin efficacy, likely due to their role in renal clearance and insulin sensitivity [29, 30]. The C11orf65 rs11212617 SNP has also shown associations with improved HbA1c, fasting glucose, and postprandial glucose (PPG) levels [31]. Heritability estimates suggest that up to 30% of metformin response variability may be genetically determined [24], reinforcing the relevance of pharmacogenetic testing in precision medicine approaches to T2DM [32, 33].

Additional loci such as CAPN10 rs3792269 [34], PPARGC1A rs10213440 [35], and LOC105371611 rs12752688 [36] have been linked to improved glycemic outcomes, whereas FMO1 rs13376631 showed no significant association [37]. Collectively, these findings support the clinical potential of pharmacogenetic profiling to optimize metformin therapy and guide individualized diabetes management.

While previous studies have examined pharmacogenetic predictors of metformin efficacy primarily in patients with T2DM, few have evaluated diverse metabolic phenotypes, such as patients with MetS alone or with overlapping T2DM and MetS. Furthermore, there is a lack of region-specific data, particularly from Central Asia, on the genetic determinants of metformin response and treatment tolerability.

This study is aimed at evaluating the clinical and biochemical response to metformin therapy across different metabolic phenotypes—specifically among individuals with MetS, T2DM, and combined MetS + T2DM—in a Kazakh population. Additionally, it seeks to analyze the association between selected genetic polymorphisms and metformin treatment outcomes, specifically efficacy (as measured by HbA1c and metabolic parameters) and the occurrence of dyspeptic side effects. By integrating genetic and phenotypic data, the study intends to contribute to the development of more personalized and effective treatment strategies for patients with metabolic disorders in Kazakhstan.

## 2. Methods

### 2.1. Study Design and Population

This was a prospective cohort study conducted in Kazakhstan. Ethnicity was determined through individual interviews, and all participants in this study self-identified as Kazakh. A total of 206 patients aged 45–74 years who were prescribed metformin therapy were enrolled from the City Polyclinic No. 4 and No. 7.

This study was conducted in accordance with the ethical principles outlined in the Declaration of Helsinki and national regulations governing biomedical research. The study protocol was reviewed and approved by the Local Ethics Committee of Astana Medical University (Protocol No. 3, approved on 24.12.2015). All participants provided written informed consent prior to enrollment. Confidentiality and anonymity of participant data were strictly maintained throughout the study. Participation was entirely voluntary, and individuals retained the right to withdraw at any stage without consequences.

Participants were stratified into three clinical groups based on their initial diagnosis: (1) MetS (*n* = 44), (2) MetS with T2DM (MetS + T2DM; *n* = 123), and (3) T2DM without MetS (T2DM; *n* = 39). Group stratification was performed at baseline to reflect common real-world clinical phenotypes and assess outcomes across varying degrees of metabolic dysfunction. Eligible participants were adults aged over 18 years with a confirmed diagnosis of MetS and/or T2DM who were initiating metformin therapy.

The inclusion criteria differed slightly by group: The MetS group included individuals meeting the International Diabetes Federation (IDF) [38] criteria for MetS but without T2DM, the T2DM group included patients newly diagnosed with T2DM based on WHO criteria but not fulfilling MetS criteria, and the MetS + T2DM group included patients who met both IDF and WHO criteria [39, 40]. All patients were newly diagnosed with MetS and/or T2DM at the time of enrollment and had not previously received metformin or other glucose-lowering therapy. The term “initiating therapy” refers to the first prescription of metformin as part of standard care for newly diagnosed individuals. Lifestyle factors such as diet, physical activity, and medication adherence were managed according to national clinical protocols developed by the Ministry of Healthcare of the Republic of Kazakhstan. General practitioners provided standardized lifestyle counseling and monitored adherence throughout the follow-up period.

Initial group assignment included 50 patients with newly diagnosed MetS, of whom 6 were excluded due to the identification of acute or chronic concomitant diseases, including infectious inflammatory processes. The second group originally included 125 patients with concurrent MetS and T2DM; 2 individuals were excluded due to diabetes-related complications identified during clinical evaluation. The third group initially enrolled 50 patients with newly diagnosed T2DM without evidence of MetS or complications; however, 10 patients required escalation to multicomponent therapy and were excluded from further analysis.

MetS was diagnosed in accordance with the IDF criteria (2005), requiring central obesity (waist circumference [WC] > 94 cm in men and > 80 cm in women and/or body mass index [BMI] > 30 kg/m^2^) plus any two of the following: elevated triglycerides (≥ 1.7 mmol/L or specific lipid-lowering treatment), reduced HDL cholesterol (< 1.03 mmol/L in men and < 1.29 mmol/L in women or specific therapy), elevated blood pressure (systolic ≥ 130 mmHg or diastolic ≥ 85 mmHg, or antihypertensive treatment), and elevated fasting plasma glucose (FPG) (≥ 5.6 mmol/L) or previously diagnosed Type 2 diabetes [39].

The diagnosis of T2DM was based on the WHO criteria: FPG ≥ 7.0 mmol/L, 2-h postload glucose ≥ 11.1 mmol/L after a 75 g oral glucose tolerance test, or HbA1c ≥ 6.5% [40].

Exclusion criteria included severe or decompensated diabetes, anemia, ischemic heart disease, chronic heart failure, chronic renal failure, or acute and decompensated comorbidities including infectious diseases within the preceding 6 months.

### 2.2. Clinical and Biochemical Assessment

Anthropometric parameters (weight, BMI, and WC) and hemodynamic measurements (systolic and diastolic blood pressure [SBP and DBP]) were recorded at baseline and after 3 months of metformin therapy. Biochemical parameters, including FPG, PPG, HbA1c, and total cholesterol, were assessed using standard clinical laboratory methods.

Determination of glucose content in venous plasma was performed on an empty stomach and 2 h after a meal using the acoustic analysis method on the COBAS C411 analyzer (Roche Diagnostics International AG, Rotkreuz, Switzerland). To determine the level of glycosylated hemoglobin, the turbodimetric method of the immunofluorescence analyzer COBAS INTEGRA 400 plus (Roche Diagnostics International AG, Rotkreuz, Switzerland), certified in accordance with NGSP, certified in accordance with DCCT reference values, was used.

To analyze lipid metabolism, a comprehensive lipidogram was studied with determination of the content of total cholesterol, triglycerides, and high-density lipoprotein cholesterol in the blood serum, which were determined using the COBAS analyzer (Roche Diagnostics International AG, Rotkreuz, Switzerland).

Metformin intolerance was assessed by the development of the following side effects: hypoglycemia, dyspepsia symptoms (bloating, nausea after eating, flatulence, and diarrhea), and hepatalgia. Patients were called 2 weeks after starting metformin, and those who responded positively to the development of side effects were excluded from the study.

### 2.3. Treatment and Dosage

Patients received individualized metformin dosages based on Clinical Protocols of the Ministry of Healthcare of the Republic of Kazakhstan “Diabetes mellitus type 2” (No. 158 dated 04.03.2022) and “Obesity in adults” (No. 196 dated 07.12.2023). Average daily metformin doses were recorded, and dosage differences between groups were analyzed. On average, individuals with MetS were prescribed lower daily doses (approximately 989 ± 347 mg), while those with MetS + T2DM and T2DM received higher doses, averaging 1461 ± 405 mg and 1346 ± 476 mg, respectively.

Treatment efficacy was evaluated using two definitions:

• Treatment Efficacy-1 for the whole three-group patients: categorized HbA1c reduction (< 0.5%, > 0.5%, or no effect) [41]. This type of efficacy was applied, taking into account the first group of patients with MetS without T2DM, given that their initial level of HbA1c was less than 6.5%.
• Treatment Efficacy-2: achievement of target glycemic control, based on the presence or absence of atherosclerotic cardiovascular disease and/or risk of severe hypoglycemia according to Clinical Protocol of the Ministry of Healthcare of the Republic of Kazakhstan “Diabetes mellitus type 2” (No. 158 dated 04.03.2022). This type of efficiency was used to compare the results between two groups: the second group (MetS + T2DM) and the third group (T2DM).

### 2.4. Genotyping

DNA was extracted using the salting out method [42]. The concentration and purity of DNA were determined spectrophotometrically using the NanoDrop 1000 Spectrophotometer (Thermo Fisher Scientific Inc., Waltham, Massachusetts, United States). A total of 10 SNPs previously implicated in metformin pharmacogenetics were selected based on existing literature. These included variants in genes encoding organic cation transporters (e.g., SLC22A1, SLC22A2, SLC22A3, SLC47A1, and SLC47A2), metabolism-related genes (FMO1 and PPARGC1A), and regulatory regions (C11orf65, CAPN10, and LOC105371611), which have been associated with glycemic response and metformin tolerance in prior studies [27–29, 36, 37, 43–49].

Genotyping of selected SNPs (listed in Table S1) was performed using predesigned TaqMan SNP Genotyping Assays (Life Technologies, Foster City, California, United States). Analyses were conducted according to the manufacturer's standard protocols. Data analyses were performed using TaqMan Genotyper Software V1.3.

In the first group (MetS), 44 samples were taken, of which 1 sample was of unsatisfactory quality and 43 samples were genotyped. In the second group (MetS + T2DM), 123 samples were taken and genotyped. In the third group (T2DM), 40 samples were taken, of which 1 sample was of unsatisfactory quality and 39 samples were genotyped.

### 2.5. Statistical Analysis

Descriptive statistics, including means, standard deviations, and frequencies, were calculated for baseline and follow-up clinical parameters. Changes in clinical and biochemical parameters before and after treatment were analyzed using repeated measures analysis of variance (ANOVA), with time as a within-subject factor, clinical group as a between-subject factor, and metformin dosage as a covariate. The conformance of genotype frequency distributions to the Hardy–Weinberg equilibrium (HWE) was assessed using the *χ*^2^ criterion (*α* = 0.05, df = 2). To assess the effects of genetic variants on treatment response, genotype-by-time interaction effects were evaluated using repeated measures ANOVA. Associations between specific genotypes and treatment efficacy outcomes (categorized either by HbA1c reduction or achievement of glycemic targets) were analyzed using *χ*^2^ tests. Ordinal logistic regression analysis was employed to identify predictors of the degree of HbA1c reduction (Treatment Efficacy-1), while binomial logistic regression was used to identify predictors of achieving glycemic goals (Treatment Efficacy-2) and the occurrence of metformin-related dyspepsia. Genotypic associations were analyzed using a genotype model in which heterozygote and homozygote alleles were each compared separately to another homozygote allele. This approach allows for the detection of both additive and nonadditive effects. A *p* value of less than 0.05 was considered statistically significant. All statistical analyses were performed using Jamovi Version 2.6.17.

## 3. Results

A total of 206 patients receiving metformin were included in the analysis, comprising 44 with MetS, 123 with both MetS and Type 2 diabetes (MetS + T2DM), and 39 with T2DM. The average age was comparable across groups: 58.7 ± 8.84 years in the MetS group, 58.4 ± 9.57 years in the MetS + T2DM group, and 59.4 ± 8.49 years in the T2DM group. ANOVA confirmed no significant differences in age between groups (*p* = 0.831). However, gender distribution differed significantly, with males representing 61.5% in the T2DM group, 39.8% in the MetS + T2DM group, and 34.1% in the MetS group (*χ*^2^ = 7.40, *p* = 0.025).

Metformin treatment was associated with significant improvements in anthropometric, hemodynamic, and biochemical parameters in all groups. After 3 months of treatment, significant reductions were observed across most clinical and biochemical parameters in all groups (Table 1). Notably, HbA1c levels decreased significantly in the MetS and MetS + T2DM groups but not in the T2DM group (*p* = 0.054). Additionally, total cholesterol levels declined significantly in all groups. Full results are summarized in Table 1.

When evaluating treatment efficacy, the distribution of HbA1c response using the three-category scale of Treatment Efficacy-1 (no effect, < 0.5% reduction, and > 0.5% reduction) did not differ significantly between the groups (Table 2).

Analysis of Treatment Efficacy-2, defined as the achievement of target glycemic control, was conducted only for patients with T2DM and MetS + T2DM. The *χ*^2^ test did not reveal a statistically significant difference in target achievement between these two groups (*χ*^2^ = 0.61, *p* = 0.433).

The average daily dosage of metformin varied significantly among the clinical groups. According to Welch's ANOVA, there was a statistically significant difference in dosage between the groups (*F* = 27.3, *p* < 0.001). To assess the effect of metformin treatment on various anthropometric, hemodynamic, and biochemical parameters, a series of repeated measures ANOVAs was conducted. The within-subject factor was time (pre- vs. posttreatment), while clinical group (MetS, MetS + T2DM, and T2DM) was included as a between-subject factor and metformin dosage as a covariate. The analysis is aimed at determining whether treatment effects differed across clinical groups and whether metformin dosage moderated these effects (Table S2).

Significant main effects of time were observed for nearly all outcomes, indicating improvements from baseline to posttreatment. No statistically significant time-by-group interactions were found for any parameter (*p* > 0.05), indicating that changes over time were generally consistent across the three clinical groups. When metformin dosage was included as a covariate, a significant time-by-dosage interaction was detected only for HbA1c (*p* = 0.029, *η*^2^ = 0.037), suggesting that higher or lower dosages may have moderated the treatment effect on glycemic control. No other outcomes demonstrated significant interactions with dosage, although small effect sizes were noted for weight, FPG, and cholesterol (all *η*^2^ < 0.012). These findings suggest that metformin was effective in improving key clinical and metabolic indicators regardless of group assignment, with minimal moderation by dosage except in the case of HbA1c.

The distribution of genotypes and allele frequencies was analyzed for 10 selected SNPs associated with metformin pharmacogenetics (Table S3). Across the sample, most genotype distributions were consistent with known population-level frequencies from dbSNP. No statistically significant intergroup differences were found for any SNPs after chi-square testing (*p* > 0.05), although rs11212617 showed a borderline association across groups (*p* = 0.05).

To assess the influence of individual genetic variants on treatment outcomes, repeated measures ANOVA was conducted across the entire sample, independent of clinical group. Each analysis examined the interaction between time (before vs. after treatment) and genotype for a given SNP in relation to multiple clinical and biochemical parameters.  Table 3 presents the *F* values and corresponding significance levels for these interactions.

Significant genotype-dependent changes over time were observed for rs2289669 and rs3792269 across multiple parameters, including weight, BMI, WC, SBP and DBP, FPG, PPG, HbA1c, and total cholesterol. Additionally, rs13376631 showed significant interactions with DBP, fasting and PPG, HbA1c, and total cholesterol. Other SNPs demonstrated isolated associations with specific parameters but did not show consistent interaction patterns. Overall, rs2289669 and rs3792269 emerged as the most influential variants modulating treatment-related clinical and metabolic changes.

Associations between SNPs and clinical treatment efficacy were evaluated using *χ*^2^ square tests. Among the SNPs analyzed, three showed significant associations with Treatment Efficacy-1 (defined by change in HbA1c levels). The genotype distribution of rs2289669 was strongly associated with Treatment Efficacy-1 (*χ*^2^ = 31.9, *p* < 0.001), followed by significant associations for rs3792269 (*χ*^2^ = 13.0, *p* = 0.011) and rs11212617 (*χ*^2^ = 9.91, *p* = 0.042).

To identify predictors of treatment efficacy categorized by HbA1c reduction (Treatment Efficacy-1), an ordinal logistic regression analysis was performed. The overall model was statistically significant (*χ*^2^ = 37.4, *p* < 0.001), explaining approximately 11.7% of the variance in treatment efficacy (Table 4).

Although rs3792269 showed a significant association with HbA1c reduction (Table 3), it was excluded from the ordinal logistic regression model due to insufficient observations in specific genotype categories (particularly the GG genotype, which had only three cases). This limitation could lead to model instability, convergence issues, or inflated standard errors. Therefore, to maintain the robustness and reliability of the regression model, only SNPs with adequate genotype distribution (such as rs11212617 and rs2289669) were included in the final ordinal logistic regression analysis.

Among clinical variables, metformin dosage was a significant predictor. Higher dosages were associated with a lower likelihood of achieving greater HbA1c reduction (OR = 0.999, *p* = 0.004), suggesting that patients requiring higher doses tended to have a less favorable treatment response. Other clinical factors, including age, gender, BMI, baseline HbA1c, and clinical group, were not significant predictors of Treatment Efficacy-1.

Regarding genetic factors, rs2289669 demonstrated a strong association with treatment efficacy. Carriers of the AG genotype had significantly lower odds of achieving greater HbA1c reductions compared to AA carriers (OR = 0.161, *p* < 0.001). Similarly, individuals with the GG genotype also exhibited reduced odds of achieving treatment success (OR = 0.139, *p* < 0.001). Genotypes of rs11212617 were not significantly associated with treatment efficacy after adjustment for clinical covariates.

It should be noted that rs3792269 initially demonstrated a significant association with Treatment Efficacy-1 in the independent *χ*^2^ square analysis; however, due to insufficient observations within specific genotype categories, this SNP was excluded from the final ordinal logistic regression model to ensure model stability and avoid convergence issues. These findings suggest that both metformin dosage and rs2289669 genotype significantly influence the likelihood of achieving substantial HbA1c reduction with metformin therapy.

For Treatment Efficacy-2 (defined as achieving target glycemic control among the T2DM and MetS + T2DM groups), significant associations were also observed. Genotype of rs2289669 was significantly associated with goal achievement (*χ*^2^ = 9.87, *p* = 0.007), as was rs3792269 (*χ*^2^ = 10.2, *p* = 0.005). These results suggest that genetic variation at these loci may influence metformin response and treatment success in T2DM patients with or without MetS.

To identify predictors of achieving target glycemic control (Treatment Efficacy-2) among patients with T2DM with or without concurrent MetS, a binomial logistic regression analysis was conducted. The model was statistically significant (*χ*^2^ = 66.0, *p* < 0.001) and explained approximately 45.2% of the variance in treatment success (Table 5).

Among clinical variables, age emerged as a significant positive predictor of achieving glycemic targets (OR = 1.134, *p* = 0.001), indicating that older patients had a higher likelihood of treatment success. Additionally, metformin dosage was negatively associated with treatment efficacy (OR = 0.996, *p* < 0.001). Other clinical factors, including gender, BMI, baseline HbA1c, and group (MetS + T2DM vs. T2DM), were not significantly associated with Treatment Efficacy-2 in the adjusted model.

Regarding genetic predictors, rs2289669 was strongly associated with treatment outcome. Individuals carrying the AG genotype had significantly reduced odds of achieving glycemic control compared to AA carriers (OR = 0.048, *p* < 0.001). The GG genotype of rs2289669 showed a similar trend but did not reach conventional statistical significance (*p* = 0.060). In addition, carriers of the AG genotype of rs3792269 also demonstrated significantly lower odds of achieving glycemic targets compared to AA homozygotes (OR = 0.125, *p* = 0.021). The GG genotype of rs3792269 could not be reliably interpreted due to convergence issues. These findings highlight the important role of age and specific genetic variations in predicting metformin treatment success among patients with T2DM, with or without concurrent MetS.

To explore genetic factors associated with metformin-related side effects, we examined the presence of dyspepsia in relation to SNP genotype using chi-square analysis. Among the tested variants, two SNPs demonstrated significant associations with reported gastrointestinal intolerance. The genotype distribution of rs2289669 was significantly associated with dyspepsia occurrence (*χ*^2^ = 12.8, *p* = 0.002), and a highly significant association was also found for rs3792269 (*χ*^2^ = 59.0, *p* < 0.001). No other SNPs showed a statistically significant relationship with dyspepsia. These findings suggest a potential pharmacogenetic basis for adverse effects, particularly involving genetic variation in rs2289669 and rs3792269.

To identify clinical and genetic predictors of metformin-associated dyspepsia, a binomial logistic regression analysis was conducted. The overall model was statistically significant (*χ*^2^ = 63.4, *p* < 0.001), with a McFadden's *R*^2^ of 0.499, indicating that approximately 50% of the variance in dyspepsia occurrence was explained by the included predictors (Table 6).

Among all covariates, only genetic variation at rs3792269 was significantly associated with the risk of dyspepsia. Specifically, individuals carrying the AG genotype had significantly higher odds of experiencing dyspepsia compared to AA carriers (OR = 59.17, *p* < 0.001). The GG genotype of rs3792269 was included but yielded an unstable estimate and was not statistically significant. Although rs2289669 showed strong associations in unadjusted analyses, its adjusted odds ratios were extreme and statistically nonsignificant, likely due to sparse data or multicollinearity. No significant associations were observed for clinical group, age, sex, baseline BMI, and HbA1c, or metformin dosage in this model. These findings suggest a potential pharmacogenetic susceptibility to gastrointestinal side effects associated with the AG genotype of rs3792269, independent of clinical and demographic factors.

## 4. Discussion

This study demonstrated that metformin therapy significantly improved anthropometric, biochemical, and hemodynamic parameters in all three patient groups—those with MetS, T2DM, and combined MetS + T2DM. Notably, reductions in HbA1c were observed across all groups, although the magnitude of glycemic response varied by clinical phenotype. Importantly, the therapeutic efficacy of metformin was influenced by specific genetic variants, particularly rs2289669 in the SLC47A1 gene and rs3792269 in CAPN10, which were associated with improved glycemic control. Additionally, several pharmacogenetic markers were linked not only to treatment response but also to the occurrence of metformin-induced dyspepsia, highlighting their potential role in guiding both efficacy and tolerability-based patient stratification. These findings underscore the relevance of integrating genetic information into the clinical management of metabolic disorders.

To our knowledge, this is the first study to comprehensively examine the clinical response and pharmacogenetic predictors of metformin therapy in a Kazakh population. While previous studies in Korean, Indian, Chinese, and European cohorts have highlighted the relevance of SLC22A1, SLC47A1, and CAPN10 polymorphisms in metformin response [29, 43, 46, 47], our findings extend this body of evidence to Central Asia and demonstrate consistency in the impact of these variants across diverse ethnic groups. By integrating clinical and genetic data, this study contributes to the growing field of precision medicine in metabolic disease and underscores the importance of population-specific research in informing individualized treatment strategies.

### 4.1. Clinical Implications of Metformin Response

Metformin remains the cornerstone of pharmacological therapy for T2DM due to its well-documented efficacy in improving glycemic control, favorable safety profile, and additional metabolic benefits [50–52]. In our study, metformin treatment resulted in meaningful reductions in HbA1c across all groups. The average absolute HbA1c reduction was 0.469% in the MetS group (partial *η*^2^ = 0.710), 0.474% in the MetS + T2DM group (partial *η*^2^ = 0.490), and 0.288% in the T2DM group (partial *η*^2^ = 0.144). While the reduction in the T2DM group showed a borderline trend (*p* = 0.054), the effect sizes observed in the MetS and MetS + T2DM groups were large to moderate, suggesting clinically significant improvements in glycemic control even with short-term metformin use. These findings are consistent with prior evidence showing metformin's ability to lower blood glucose without promoting weight gain or causing hypoglycemia [53, 54] and support its broader application in earlier stages of metabolic dysfunction.

Beyond glycemic control, metformin has been shown to confer cardiovascular benefits by lowering blood pressure, improving lipid profiles, and enhancing endothelial function [51, 53, 54]. Our cohort also demonstrated improvements in SBP and DBP, body weight, and total cholesterol following metformin therapy, reinforcing its role in mitigating cardiometabolic risk. In the subgroup with MetS alone, metformin improved insulin sensitivity and modestly reduced weight—effects that align with previous studies emphasizing its utility in managing hyperinsulinemia and obesity in normoglycemic individuals [20, 53]. Although the evidence remains mixed regarding its efficacy in patients without overt hyperglycemia, our results suggest that metformin may offer preventive benefits in such high-risk populations, especially when combined with lifestyle modification [20, 51, 53].

Taken together, these findings highlight metformin's broad therapeutic potential across the metabolic disease spectrum and support its continued use as a first-line agent not only in T2DM but also in individuals with MetS and high cardiometabolic risk.

### 4.2. Pharmacogenetic Insights

Our study identified significant associations between metformin treatment outcomes and specific genetic polymorphisms, supporting previous evidence that pharmacogenetics plays a critical role in modulating drug response in individuals with metabolic disorders. Notably, the SLC47A1 rs2289669 variant demonstrated a strong association with glycemic response: Individuals carrying the AA genotype exhibited a significantly greater reduction in HbA1c compared to GA and GG genotypes. This was confirmed in both repeated measures ANOVA (Table 3) and ordinal logistic regression (Table 4), where GA and GG genotypes were associated with markedly lower odds of achieving a > 0.5% reduction in HbA1c. These findings align with prior studies reporting that homozygous A allele carriers experience nearly twice the HbA1c reduction relative to G allele carriers [43, 55, 56].

Similarly, the CAPN10 rs3792269 variant was significantly associated with reduced metformin efficacy. Although excluded from the ordinal regression due to low homozygote counts, binomial logistic regression (Table 5) showed that individuals with the AG genotype had significantly lower odds of achieving glycemic targets (Treatment Efficacy-2). These results echo the findings by Tkáč et al. [43], who reported an odds ratio of 0.27 for treatment success and a smaller absolute HbA1c reduction among G allele carriers. This suggests a clinically meaningful impact of CAPN10 variation on glycemic response.

In addition to efficacy, we examined the role of these variants in metformin-associated dyspepsia, a key tolerability concern. In Table 6, the rs3792269 AG genotype was associated with a substantially increased risk of dyspeptic symptoms (OR = 59.17, *p* < 0.001), even after adjustment for clinical covariates. While rs2289669 also showed an unadjusted association, it did not retain significance in multivariable models, possibly due to sample size limitations or collinearity.

Recent research further supports the mechanistic relevance of these findings. SLC47A1 encodes the MATE1 transporter, which is crucial for renal and hepatic clearance of metformin. Polymorphisms such as rs2289669 and rare variants like L125F may impair metformin efflux, increasing systemic exposure and influencing efficacy or side effects [57, 58]. Additionally, metformin may upregulate MATE1 expression via a PPAR-*α* pathway, modulating its own transport dynamics [59]. CAPN10 encodes calpain-10, a protease involved in insulin secretion and glucose uptake. The rs3792269 variant has been associated with differential glycemic response across populations, likely due to its role in modulating *β*-cell function and insulin sensitivity [34]. Together, these findings reinforce that both metformin pharmacokinetics (via SLC47A1) and pharmacodynamics (via CAPN10) are subject to genetic regulation, supporting genotype-guided therapy in metabolic disease.

Although our study did not observe a significant effect of rs11212617 on HbA1c reduction in isolation, previous meta-analyses involving European populations have consistently demonstrated a modest but significant association between this variant and treatment success [60, 61]. While the precise mechanisms remain unclear, this locus, near the ATM gene, may influence metformin's action via pathways related to cellular stress responses or insulin signaling.

Taken together, these results underscore the importance of genetic variation in both pharmacokinetic (SLC47A1) and pharmacodynamic (CAPN10) pathways in determining metformin response. The inclusion of rs2289669 and rs3792269 in pharmacogenetic screening panels could improve early prediction of treatment outcomes and guide more individualized therapy, particularly in settings like Kazakhstan, where pharmacogenetic data have been limited.

### 4.3. Strengths and Limitations

A key strength of this study lies in its prospective design and systematic collection of clinical, biochemical, and genetic data. The stratification of participants into distinct metabolic phenotypes (MetS, T2DM, and MetS + T2DM) allowed for nuanced analysis of metformin response across the metabolic spectrum. Moreover, the inclusion of a focused genotype-wide analysis, particularly of known pharmacogenetic markers, adds substantial value to the growing body of literature on personalized diabetes care.

However, several limitations should be acknowledged. The study population was limited to individuals of Kazakh ethnicity, which, while valuable for generating region-specific insights, may limit the generalizability of findings to other ethnic groups. The follow-up duration was relatively short, restricting the evaluation of long-term treatment effects and the sustainability of glycemic control. Additionally, potential confounding factors such as diet, physical activity, and medication adherence were not controlled or systematically recorded, which may have influenced treatment outcomes.

### 4.4. Implications for Practice and Future Research

The findings of this study underscore the clinical relevance of pharmacogenetic profiling in predicting metformin efficacy and tolerability. The significant associations identified between genetic variants (e.g., SLC47A1 rs2289669 and CAPN10 rs3792269) and HbA1c reduction suggest that genotyping could be a valuable tool in guiding personalized metformin therapy. Integrating such approaches into routine clinical practice may improve treatment outcomes and reduce unnecessary trial-and-error prescribing.

To validate and expand on these findings, future studies should aim at including larger and ethnically diverse cohorts, as well as longer follow-up periods. Replication across multiple populations will be essential for confirming the predictive value of specific polymorphisms. Importantly, our results advocate for the incorporation of pharmacogenetic screening into national diabetes care protocols in Kazakhstan, aligning with broader precision medicine initiatives and contributing to more effective, individualized management of metabolic disorders.

While our findings highlight the clinical value of pharmacogenetic testing for metformin response, its implementation in Kazakhstan faces practical challenges. Currently, such testing is not part of routine care and remains limited to research initiatives [62]. Costs vary widely, ranging from ~$75 to several hundred USD per test, which may be prohibitive in local settings without insurance coverage [32, 63, 64]. To enable real-world integration, affordable testing options and streamlined infrastructure will be essential. Additionally, future research should assess the cost-effectiveness of pharmacogenetic screening in the context of MetS and T2DM care in Kazakhstan.

Moreover, future studies should include longer follow-up to assess the durability of glycemic response and genetic associations beyond 3 months. We also observed dosage-related differences in HbA1c outcomes; however, since dosing was not randomized, further research is needed to determine whether these differences reflect true pharmacogenetic effects or underlying clinical severity.

## 5. Conclusion

This study demonstrates that metformin therapy significantly improves clinical and biochemical parameters in Kazakh patients with MetS, T2DM, and their combination. Importantly, individual treatment responses and adverse effects were strongly influenced by genetic polymorphisms, particularly rs2289669 in SLC47A1 and rs3792269 in CAPN10. These findings underscore the clinical relevance of pharmacogenetic profiling for predicting both efficacy and tolerability of metformin. As the first study of its kind in Kazakhstan, it highlights the need to integrate genetic screening into national diabetes management protocols and supports the advancement of personalized medicine in Central Asia. Further studies in larger, multiethnic cohorts are warranted to validate these findings and facilitate broader clinical application.

## Figures and Tables

**Table 1:** Clinical and biochemical parameters before and after metformin treatment in patients with metabolic syndrome (MetS), Type 2 diabetes mellitus (T2DM), and combined MetS + T2DM.

**Variable**	**MetS ( ** **n** = 44** )**			**MetS + T2DM ( ** **n** = 123** )**			**T2DM ( ** **n** = 39** )**		
	**Before treatment**	**After treatment**	**p**	**Before treatment**	**After treatment**	**p**	**Before treatment**	**After treatment**	**p**
Weight (kg)	90.5 ± 9.02	86.2 ± 9.80	< 0.001	91.9 ± 11.74	87.4 ± 12.26	< 0.001	76.8 ± 8.74	72.9 ± 8.93	< 0.001
BMI (kg/m^2^)	33.5 ± 2.91	31.9 ± 3.08	< 0.001	33.5 ± 3.08	31.9 ± 3.37	< 0.001	27.4 ± 1.38	26.1 ± 1.57	< 0.001
WC (cm)	104.2 ± 8.06	101.0 ± 8.42	< 0.001	105.0 ± 10.08	101.6 ± 10.54	< 0.001	84.6 ± 7.84	81.7 ± 8.19	< 0.001
SBP (mmHg)	128 ± 11.9	121 ± 11.7	< 0.001	130 ± 13.4	123 ± 11.0	< 0.001	129 ± 15.0	120 ± 12.1	< 0.001
DBP (mmHg)	81.7 ± 9.27	76.1 ± 6.81	< 0.001	81.7 ± 8.07	77.2 ± 7.08	< 0.001	81.7 ± 8.14	76.4 ± 7.43	< 0.001
FPG	7.20 ± 0.54	6.00 ± 0.87	< 0.001	8.61 ± 0.98	7.48 ± 1.47	< 0.001	8.73 ± 0.91	7.63 ± 1.25	< 0.001
PPG	8.87 ± 0.75	7.82 ± 0.91	< 0.001	10.66 ± 1.55	9.39 ± 1.73	< 0.001	10.86 ± 1.62	9.57 ± 1.51	< 0.001
HbA1C (%)	6.26 ± 0.15	5.80 ± 0.33	< 0.001	7.24 ± 0.34	6.77 ± 0.54	< 0.001	7.28 ± 0.35	6.99 ± 0.83	0.054
Total cholesterol	6.71 ± 0.75	5.57 ± 1.03	< 0.001	6.89 ± 0.95	5.81 ± 1.17	< 0.001	6.26 ± 0.81	5.19 ± 1.28	< 0.001

*Note:* Data are presented as mean ± standard deviation (SD).

Abbreviations: BMI, body mass index; DBP, diastolic blood pressure; FPG, fasting plasma glucose; HbA1c, glycated hemoglobin; PPG, postprandial glucose; SBP, systolic blood pressure; WC, waist circumference.

**Table 2:** Distribution of treatment efficacy across clinical groups based on HbA1c reduction (Treatment Efficacy-1).

**Group**	**Treatment Efficacy-1**			**χ** ^2^, **p**
	**No effect**	**< 0.5%**	**> 0.5%**	
MetS	4 (9.1%)	21 (47.7%)	19 (43.2%)	1.96, 0.743
MetS + T2DM	19 (15.4%)	52 (42.3%)	52 (42.3%)	
T2DM	6 (15.4%)	14 (35.9%)	19 (48.7%)	

*Note:* Values represent the number of patients (*n*) and percentage within each group (%). Statistical comparison between groups was performed using the chi-square (*χ*^2^) test.

Abbreviations: HbA1c, glycated hemoglobin; MetS, metabolic syndrome; T2DM, Type 2 diabetes mellitus.

**Table 3:** Time and genotype interaction effects (*F* values) on clinical and biochemical outcomes in the whole sample.

**SNPs**	**Weight**	**BMI**	**WC**	**SBP**	**DBP**	**FPG**	**PPG**	**HbA1C**	**Total cholesterol**
rs13376631	3.25⁣^∗^	2.95	2.52	1.72	4.15⁣^∗^	3.10⁣^∗^	3.82⁣^∗^	7.30⁣^∗∗∗^	4.27⁣^∗^
rs12943590	0.79	0.55	0.45	0.20	0.69	1.38	2.95	2.29	7.46⁣^∗∗∗^
rs12752688	1.51	2.26	1.67	1.72	0.93	2.26	1.72	1.12	3.11⁣^∗^
rs12208357	0.84	1.13	0.19	0.01	0.00	0.11	0.81	0.10	4.53⁣^∗^
rs10213440	0.41	0.24	0.91	0.32	0.31	0.41	0.35	0.12	0.305
rs2289669	38.9⁣^∗∗∗^	36.2⁣^∗∗∗^	29.7⁣^∗∗∗^	8.98⁣^∗∗∗^	4.66⁣^∗^	13.7⁣^∗∗∗^	19.2⁣^∗∗∗^	7.36⁣^∗∗∗^	170⁣^∗∗∗^
rs3792269	55.7⁣^∗∗∗^	50.4⁣^∗∗∗^	70.0⁣^∗∗∗^	35.3⁣^∗∗∗^	37.4⁣^∗∗∗^	6.76⁣^∗∗^	6.88⁣^∗∗^	3.78⁣^∗^	8.35⁣^∗∗∗^
rs2076828	1.33	1.12	1.14	0.46	0.69	0.68	0.22	0.44	1.71
rs11212617	0.70	1.37	0.47	0.17	0.02	1.70	2.92	2.79	4.33⁣^∗^
rs594709	1.05	0.362	1.02	0.36	0.85	1.36	0.39	3.07⁣^∗^	0.539

*Note:* Values represent *F* values from repeated measures ANOVA, indicating the interaction effect of time and SNP genotype. Significant values are denoted as ⁣^∗^*p* < 0.05, ⁣^∗∗^*p* < 0.01, and ⁣^∗∗∗^*p* < 0.001. Analyses were not stratified by clinical group.

Abbreviations: BMI, body mass index; DBP, diastolic blood pressure; FPG, fasting plasma glucose; HbA1c, glycated hemoglobin; PPG, postprandial glucose; SBP, systolic blood pressure; SNP, single nucleotide polymorphism; WC, waist circumference.

**Table 4:** Ordinal logistic regression predicting Treatment Efficacy-1 from clinical and genetic factors.

**Predictor**	**Beta**	**SE**	**OR**	**OR 95% CI**	**p**
Group					
MetS + T2DM–MetS	0.186	0.294	1.205	—	0.526
T2DM–MetS	0.383	0.206	1.468	—	0.062
Age	−0.005	0.016	0.995	0.960–1.030	0.751
Gender					
Female–male	0.347	0.334	1.415	0.734–2.737	0.299
Metformin dosage	−0.001	0.0005	0.999	0.998–0.999	0.004
BMI before treatment	0.059	0.038	1.060	0.934–1.209	0.124
HbA1C before treatment	0.292	0.201	1.339	—	0.146
rs2289669					
AG–AA	−1.824	0.400	0.161	0.071–0.348	< 0.001
GG–AA	−1.976	0.542	0.139	0.046–0.397	< 0.001
rs11212617					
AC–AA	0.422	0.419	1.526	0.621–3.753	0.314
CC–AA	0.458	0.434	1.581	0.609–4.123	0.291

*Note:* Model statistics: *χ*^2^ = 37.4, *p* < 0.001, *R*^2^_McF_ = 0.117. Treatment Efficacy-1 was defined as HbA1c reduction categorized into no effect, < 0.5%, and > 0.5%.

Abbreviations: BMI, body mass index; CI, confidence interval; HbA1c, glycated hemoglobin; MetS, metabolic syndrome; OR, odds ratio; SE, standard error; T2DM, Type 2 diabetes mellitus.

**Table 5:** Binomial logistic regression predicting glycemic goal achievement (Treatment Efficacy-2) in patients with Type 2 diabetes mellitus with or without metabolic syndrome.

**Predictor**	**Beta**	**SE**	**OR**	**OR 95% CI**	**p**
Group					
MetS + T2DM–T2DM	0.750	0.952	2.117	0.328–13.680	0.431
Age	0.126	0.039	1.134	1.052–1.224	0.001
Gender					
Female–male	0.611	0.587	1.842	0.583–5.817	0.298
Metformin dosage	−0.004	0.001	0.996	0.995–0.998	< 0.001
BMI before treatment	0.110	0.106	1.116	0.906–1.375	0.301
HbA1C before treatment	−0.823	0.820	0.439	0.088–2.188	0.315
rs2289669					
AG–AA	−3.043	0.818	0.048	0.010–0.237	< 0.001
GG–AA	−2.027	1.078	0.132	0.016–1.091	0.060
rs3792269					
AG–AA	−2.076	0.899	0.125	0.022–0.731	0.021
GG–AA	−17.280	1692.7	0.0000	0.000–Inf	0.992

*Note:* Model statistics: *χ*^2^ = 66.0, *p* < 0.001, *R*^2^_McF_ = 0.452. Treatment Efficacy-2: defined as achievement of target glycemic control based on the national clinical protocol for Type 2 diabetes mellitus (Kazakhstan, Order No. 158, 04.03.2022).

Abbreviations: BMI, body mass index; CI, confidence interval; HbA1c, glycated hemoglobin; Inf, infinity; MetS, metabolic syndrome; OR, odds ratio; SE, standard error; T2DM, Type 2 diabetes mellitus.

**Table 6:** Binomial logistic regression predicting dyspepsia (side effect) from clinical and genetic factors.

**Predictor**	**Beta**	**SE**	**OR**	**OR 95% CI**	**p**
Group					
MetS + T2DM–MetS	0.079	1.350	1.082	0.077–15.26	0.953
T2DM–MetS	0.369	1.720	1.446	0.050–42.09	0.830
Age	0.032	0.043	1.033	0.949–1.12	0.455
Gender					
Female–male	0.524	0.737	1.689	0.399–7.16	0.477
Metformin dosage	0.001	0.001	1.001	0.999–1.00	0.189
BMI before treatment	−0.065	0.127	0.937	0.730–1.20	0.609
HbA1C before treatment	−0.159	1.140	0.853	0.091–7.96	0.889
rs2289669					
AG–AA	20.884	2064.4	1.2⁣^∗^10^9^	0.000–Inf	0.992
GG–AA	19.746	2064.4	3.8⁣^∗^10^8^	0.000–Inf	0.992
rs3792269					
AG–AA	4.080	0.908	59.171	9.977–350.91	< 0.001
GG–AA	23.686	12506.4	1.9⁣^∗^10^10^	0.000–Inf	0.998

*Note:* Model statistics *χ*^2^ = 63.4, *p* < 0.001, *R*^2^_McF_ = 0.499. Dyspepsia was analyzed as a binary adverse event outcome.

Abbreviations: BMI, body mass index; CI, confidence interval; HbA1c, glycated hemoglobin; Inf, infinity; MetS, metabolic syndrome; OR, odds ratio; SE, standard error; T2DM, Type 2 diabetes mellitus.

## Data Availability

The dataset supporting the conclusions of this article is available from the corresponding author.

## Supporting Information

Supporting Information Additional supporting information can be found online in the Supporting Information section. Table S1: Characteristics of selected SNPs, including SNP ID, gene or nearby region, variant consequence, genomic position (GRCh38.p14), and literature references. Table S2: Results of repeated measures ANOVA showing the effects of time, clinical group, and metformin dosage on clinical and biochemical parameters (weight, BMI, WC, SBP, DBP, FPG, PPG, HbA1c, and total cholesterol). Values include *F*-statistics, *p* values, and partial *η*^2^. Table S3: Genotype distributions and allele frequencies of selected SNPs in the study population, with intergroup chi-square tests and comparisons against dbSNP global allele frequencies.

## References

[1] He K. J., Wang H., Xu J., Gong G., Liu X., Guan H. (2024). Global Burden of Type 2 Diabetes Mellitus From 1990 to 2021, With Projections of Prevalence to 2044: A Systematic Analysis Across SDI Levels for the Global Burden of Disease Study 2021. *Frontiers in Endocrinology*.

[2] GBD 2021 Diabetes Collaborators (2023). Global, Regional, and National Burden of Diabetes From 1990 to 2021, With Projections of Prevalence to 2050: A Systematic Analysis for the Global Burden of Disease Study 2021. *Lancet*.

[3] Khan M. A. B., Hashim M. J., King J. K., Govender R. D., Mustafa H., Al Kaabi J. (2020). Epidemiology of Type 2 Diabetes - Global Burden of Disease and Forecasted Trends. *Journal of Epidemiology and Global Health*.

[4] Ye J., Wu Y., Yang S. (2023). The Global, Regional and National Burden of Type 2 Diabetes Mellitus in the Past, Present and Future: A Systematic Analysis of the Global Burden of Disease Study 2019. *Frontiers in Endocrinology*.

[5] Getachew C. T., Hunde L., Arero G., Menberu G. (2024). Metabolic Syndrome Among Type 2 Diabetes Mellitus Patients in Ethiopia: A Systematic Review and Meta-Analysis. *Frontiers in Clinical Diabetes and Healthcare*.

[6] Saklayen M. G. (2018). The Global Epidemic of the Metabolic Syndrome. *Current Hypertension Reports*.

[7] Jamali Z., Ayoobi F., Jalali Z. (2024). Metabolic Syndrome: A Population-Based Study of Prevalence and Risk Factors. *Scientific Reports*.

[8] Zila-Velasque J. P., Grados-Espinoza P., Challapa-Mamani M. R. (2024). Prevalence of Metabolic Syndrome and Its Components According to Altitude Levels: A Systematic Review and Meta-Analysis. *Scientific Reports*.

[9] Galiyeva D., Gusmanov A., Sakko Y. (2022). Epidemiology of Type 1 and Type 2 Diabetes Mellitus in Kazakhstan: Data from Unified National Electronic Health System 2014-2019. *BMC Endocrine Disorders*.

[10] Orazumbekova B., Issanov A., Atageldiyeva K. (2022). Prevalence of Impaired Fasting Glucose and Type 2 Diabetes in Kazakhstan: Findings From Large Study. *Frontiers in Public Health*.

[11] Kazbekova A., Akanov Z., Abseitova D. (2024). The Prevalence of Diabetes Mellitus in Kazakhstan: A Systematic Review and Meta-Analysis. *Acta Biomedica Atenei Parmensis*.

[12] Ulyanova O., Karibekov T., Shaimardanova G., Kozina L. (2018). Mesenchymal Stem Cells in the Treatment of Diabetes Mellitus: Perspectives. *Journal of Clinical Medicine of Kazakhstan*.

[13] Supiyev A., Kossumov A., Kassenova A. (2016). Diabetes Prevalence, Awareness and Treatment and Their Correlates in Older Persons in Urban and Rural Population in the Astana Region, Kazakhstan. *Diabetes Research and Clinical Practice*.

[14] Helble M., Francisco K. (2017). *The Imminent Obesity Crisis in Asia and the Pacific: First Cost Estimates*.

[15] Sorokina M., Koichubekov B., Laryushina Y., Turgunova L., Bakirova R., Korshukov I. (2017). [PP.08.22] Metabolic Syndrome Prevalence Among Kazakhstan’s Population. *Journal of Hypertension*.

[16] Sadykova A., Shalkharova Z. S., Shalkharova Z. N. (2018). Metabolic Syndrome and Its Components in Southern Kazakhstan: A Cross-Sectional Study. *International Health*.

[17] Aidarbekova D., Sadykova K., Saruarov Y. (2025). A Longitudinal Assessment of Metabolic Syndrome. *Journal of Clinical Medicine*.

[18] Sibagatova A., Benberin V., Akhmetova K., Babenko D., Karabayeva R. (2024). Association of Polymorphism rs2736990 of the *α*-Synuclein Gene With Metabolic Syndrome Among the Population of Kazakhstan With Arterial Hypertension. *Metabolic Syndrome and Related Disorders*.

[19] Adeshirlarijaney A., Zou J., Tran H. Q., Chassaing B., Gewirtz A. T. (2019). Amelioration of Metabolic Syndrome by Metformin Associates With Reduced Indices of Low-Grade Inflammation Independently of the Gut Microbiota. *American Journal of Physiology. Endocrinology and Metabolism*.

[20] Kamenova P. (2020). Therapeutic Potential of Metformin in Normal Glucose Tolerant Persons With Metabolic Syndrome. *Biotechnology & Biotechnological Equipment*.

[21] Kononova Y. A., Likhonosov N. P., Babenko A. Y. (2022). Metformin: Expanding the Scope of Application-Starting Earlier Than Yesterday, Canceling Later. *International Journal of Molecular Sciences*.

[22] Park J. S., Moon S. J., Park H. S., Cho S.-H. (2024). Survival Benefit of Metformin Use According to Cancer Diagnosis in Diabetic Patients With Metabolic Syndrome. *Preventive Medicine Reports*.

[23] Aleidi S. M., Dahabiyeh L. A., Gu X. (2021). Obesity Connected Metabolic Changes in Type 2 Diabetic Patients Treated With Metformin. *Frontiers in Pharmacology*.

[24] Yee S. W., Zhou K., Giacomini K. M., Florez J. (2016). Pharmacogenetics of Metformin. *The Genetics of Type 2 Diabetes and Related Traits*.

[25] AL-Eitan L. N., Almomani B. A., Nassar A. M., Elsaqa B. Z., Saadeh N. A. (2019). Metformin Pharmacogenetics: Effects of SLC22A1, SLC22A2, and SLC22A3 Polymorphisms on Glycemic Control and HbA1c Levels. *Journal of Personalized Medicine*.

[26] Nasykhova Y. A., Barbitoff Y. A., Tonyan Z. N. (2022). Genetic and Phenotypic Factors Affecting Glycemic Response to Metformin Therapy in Patients With Type 2 Diabetes Mellitus. *Genes*.

[27] Xiao D., Guo Y., Li X. (2016). The Impacts of SLC22A1 rs594709 and SLC47A1 rs2289669 Polymorphisms on Metformin Therapeutic Efficacy in Chinese Type 2 Diabetes Patients. *International Journal of Endocrinology*.

[28] Chen E. C., Liang X., Yee S. W. (2015). Targeted Disruption of Organic Cation Transporter 3 Attenuates the Pharmacologic Response to Metformin. *Molecular Pharmacology*.

[29] He R., Zhang D., Lu W. (2015). SLC47A1 Gene rs2289669 G>A Variants Enhance the Glucose-Lowering Effect of Metformin via Delaying Its Excretion in Chinese Type 2 Diabetes Patients. *Diabetes Research and Clinical Practice*.

[30] Chen P., Cao Y., Chen S., Liu Z., Chen S., Guo Y. (2022). Association of *SLC22A1*, *SLC22A2*, *SLC47A1*, and *SLC47A2* Polymorphisms With Metformin Efficacy in Type 2 Diabetic Patients. *Biomedicine*.

[31] Zhou Y., Guo Y., Ye W. (2014). RS11212617 Is Associated With Metformin Treatment Response in Type 2 Diabetes in Shanghai Local Chinese Population. *International Journal of Clinical Practice*.

[32] Pawlyk A. C., Giacomini K. M., McKeon C., Shuldiner A. R., Florez J. C. (2014). Metformin Pharmacogenomics: Current Status and Future Directions. *Diabetes*.

[33] Damanhouri Z. A., Alkreathy H. M., Alharbi F. A., Abualhamail H., Ahmad M. S. (2023). A Review of the Impact of Pharmacogenetics and Metabolomics on the Efficacy of Metformin in Type 2 Diabetes. *International Journal of Medical Sciences*.

[34] Masilela C., Pearce B., Ongole J. J., Adeniyi O. V., Benjeddou M. (2021). Single Nucleotide Polymorphisms Associated With Metformin and Sulphonylureas’ Glycaemic Response Among South African Adults With Type 2 Diabetes Mellitus. *Journal of Personalized Medicine*.

[35] Jablonski K. A., McAteer J. B., de Bakker P. I. W. (2010). Common Variants in 40 Genes Assessed for Diabetes Incidence and Response to Metformin and Lifestyle Intervention in the Diabetes Prevention Program. *Diabetes*.

[36] Xhakaza L., Abrahams-October Z., Pearce B. (2020). Evaluation of the Suitability of 19 Pharmacogenomics Biomarkers for Individualized Metformin Therapy for Type 2 Diabetes Patients. *Drug Metabolism and Personalized Therapy*.

[37] Breitenstein M. K., Wang L., Simon G. (2015). Leveraging an Electronic Health Record-Linked Biorepository to Generate a Metformin Pharmacogenomics Hypothesis. *AMIA Joint Summits on Translational Science*.

[38] Alberti K. G., Zimmet P., Shaw J. (2006). Metabolic syndrome–a New World-Wide Definition. A Consensus Statement from the International Diabetes Federation. *Diabetic medicine: A Journal of the British Diabetic Association*.

[39] Alberti K. G., Zimmet P., Shaw J., IDF Epidemiology Task Force Consensus Group (2005). The Metabolic Syndrome--A New Worldwide Definition. *Lancet*.

[40] Alberti K. G., Zimmet P. Z. (1998). Definition, Diagnosis and Classification of Diabetes Mellitus and Its Complications. Part 1: Diagnosis and Classification of Diabetes Mellitus Provisional Report of a WHO Consultation. *Diabetic medicine: a journal of the British Diabetic Association*.

[41] Musholt P. B., Schöndorf T., Pfützner A. (2009). Combined Pioglitazone and Metformin Treatment Maintains the Beneficial Effect of Short-Term Insulin Infusion in Patients With Type 2 Diabetes: Results From a Pilot Study. *Journal of Diabetes Science and Technology*.

[42] Miller S. A., Dykes D. D., Polesky H. F. (1988). A Simple Salting Out Procedure for Extracting DNA From Human Nucleated Cells. *Nucleic Acids Research*.

[43] Tkáč I., Javorský M., Klimčáková L. (2015). A Pharmacogenetic Association Between a Variation in Calpain 10 (CAPN10) Gene and the Response to Metformin Treatment in Patients With Type 2 Diabetes. *European Journal of Clinical Pharmacology*.

[44] Zhou K., Yee S. W., Seiser E. L. (2016). Variation in the Glucose Transporter Gene *SLC2A2* Is Associated With Glycemic Response to Metformin. *Nature Genetics*.

[45] Chen P., Cao Y., Guo Y. (2022). Association of SLC22A1 rs622342 and ATM rs11212617 Polymorphisms With Metformin Efficacy in Patients With Type 2 Diabetes. *Pharmacogenetics and Genomics*.

[46] Kim J. Y., Cheong H. S., Park T. J. (2014). Screening for 392 Polymorphisms in 141 Pharmacogenes. *Biomedical Reports*.

[47] Venkatachalapathy P., Padhilahouse S., Sellappan M. (2021). Pharmacogenomics and Personalized Medicine in Type 2 Diabetes Mellitus: Potential Implications for Clinical Practice. *Pharmacogenomics and Personalized Medicine*.

[48] Stocker S. L., Morrissey K. M., Yee S. W. (2013). The Effect of Novel Promoter Variants in MATE1 and MATE2 on the Pharmacokinetics and Pharmacodynamics of Metformin. *Clinical Pharmacology and Therapeutics*.

[49] Phani N. M., Vohra M., Kakar A. (2018). Implication of Critical Pharmacokinetic Gene Variants on Therapeutic Response to Metformin in Type 2 Diabetes. *Pharmacogenomics*.

[50] Marić A. (2010). Metformin - More Than “Gold Standard” in the Treatment of Type 2 Diabetes Mellitus. *Diabetologia Croatica*.

[51] Papanas N., Maltezos E., Mikhailidis D. P. (2009). Metformin: Diamonds Are Forever. *Expert Opinion on Pharmacotherapy*.

[52] Baker C., Retzik-Stahr C., Singh V., Plomondon R., Anderson V., Rasouli N. (2021). *Should Metformin Remain the First-Line Therapy for Treatment of Type 2 Diabetes? Therapeutic Advances in Endocrinology and Metabolism*.

[53] Bailey C. J., Beck-Nielsen H. (2013). Treatment With Metformin. *The Metabolic Syndrome: Pharmacology and Clinical Aspects*.

[54] Tan M. H., Alquraini H., Mizokami-Stout K., MacEachern M. (2016). Metformin: From Research to Clinical Practice. *Endocrinology and Metabolism Clinics of North America*.

[55] Pérez-Gómez N., Fernández-Ortega M. D., Elizari-Roncal M. (2023). Identification of Clinical and Pharmacogenetic Factors Influencing Metformin Response in Type 2 Diabetes Mellitus. *Pharmacogenomics*.

[56] Mousavi S., Kohan L., Yavarian M., Habib A. (2017). Pharmacogenetic Variation of SLC47A1 Gene and Metformin Response in Type2 Diabetes Patients. *Molecular Biology Research Communications*.

[57] Avsar O. (2022). Analysis of Missense SNPs in the *SLC47A1* and *SLC47A2* Genes Affecting the Pharmacokinetics of Metformin: Computational Approach. *Egyptian Journal of Medical Human Genetics*.

[58] Morales-Rivera M. I., Alemón-Medina R., Martínez-Hernández A. (2021). The L125F MATE1 Variant Enriched in Populations of Amerindian Origin Is Associated With Increased Plasma Levels of Metformin and Lactate. *Biomedicine & Pharmacotherapy*.

[59] Arruda A. C., Perilhão M. S., Santos W. A. (2020). PPAR*α*-Dependent Modulation by Metformin of the Expression of OCT-2 and MATE-1 in the Kidney of Mice. *Molecules*.

[60] van Leeuwen N., Nijpels G., Becker M. L. (2012). A Gene Variant Near ATM Is Significantly Associated With Metformin Treatment Response in Type 2 Diabetes: A Replication and Meta-Analysis of Five Cohorts. *Diabetologia*.

[61] The GoDARTS and UKPDS Diabetes Pharmacogenetics Study Group (2011). Common Variants Near ATM Are Associated With Glycemic Response to Metformin in Type 2 Diabetes. *Nature Genetics*.

[62] Zholdybayeva E., Iskakova A., Romanova A., Ramanculov E., Momynaliev K. (2014). Pharmacogenetic Research in Kazakhstan. *Central Asian Journal of Global Health*.

[63] Maruf A. A., Fan M., Arnold P. D., Müller D. J., Aitchison K. J., Bousman C. A. (2020). Pharmacogenetic Testing Options Relevant to Psychiatry in Canada: Options de tests pharmacogénétiques pertinents en psychiatrie au Canada. *Canadian Journal of Psychiatry. Revue Canadienne de Psychiatrie*.

[64] Salas-Hernández A., Galleguillos M., Carrasco M. (2023). An Updated Examination of the Perception of Barriers for Pharmacogenomics Implementation and the Usefulness of Drug/Gene Pairs in Latin America and the Caribbean. *Frontiers in Pharmacology*.

