# Supplementary material for: Pharmacogenetic Predictors of Metformin Response in Metabolic Syndrome and Type 2 Diabetes: Evidence from a Cohort Study in Kazakhstan
Source: J Diabetes Res. 2025 Sep 26;2025:1568889. doi: 10.1155/jdr/1568889 (PMC12494468; doi:10.1155/jdr/1568889)
Supplement: Supporting Information — Additional supporting information can be found online in the Supporting Information section. Table S1: Characteristics of selected SNPs, including SNP ID, gene or nearby region, variant consequence, genomic position (GRCh38.p14), and literature references. Table S2: Results of repeated measures ANOVA showing the effects of time, clinical group, and metformin dosage on clinical and biochemical parameters (weight, BMI, WC, SBP, DBP, FPG, PPG, HbA1c, and total cholesterol). Values include F-statistics, p values, and partial η2. Table S3: Genotype distributions and allele frequencies of selected SNPs in the study population, with intergroup chi-square tests and comparisons against dbSNP global allele frequencies. [file 1568889.f1.docx]

**Supplementary materials**

**Suppl. Table 1.** Characteristic of selected SNPs.

| **SNP ID** | **Gene or nearby region** | **Consequence** | **Position**  (GRCh38.p14) | **References** |
| --- | --- | --- | --- | --- |
| rs10213440 | PPARGC1A | Intron Variant | chr4:23864716 | Tkáč et al. (2015) [36] |
| rs11212617 | C11orf65 | Intron Variant | chr11:108412434 | Zhou et al. (2016) [37]  Chen et al. (2022) [38] |
| rs12208357 | SLC22A1 | Missense Variant | chr6:160122116 | Kim et al. (2014) [39]  Venkatachalapathy et al. (2021) [40] |
| rs12752688 | LOC105371611 | Intron Variant | chr1:171182499 | Xhakaza et al. (2020) [30]  Breitenstein et al. (2015) [31] |
| rs12943590 | SLC47A2 | Intron Variant | chr17:19716685 | Stocker et al. (2013) [41]  Phani et al (2018) [42] |
| rs13376631 | FMO1 | Intron Variant | chr1:171266603 | Breitenstein et al. (2015) [31] |
| rs2076828 | SLC22A3 | 3 Prime UTR Variant | chr6:160451754 | Chen et al. (2015) [24] |
| rs2289669 | SLC47A1 | Intron Variant | chr17:19560030 | He et al. (2015) [25] |
| rs3792269 | CAPN10 | Synonymous Variant | chr2:240592062 | Tkáč et al. (2015) [36] |
| rs594709 | SLC22A1 | Intron Variant | chr6:160134722 | Xiao et al. (2016) [23] |

**Suppl. Table 2.** Effect of Time, Clinical Group, and Metformin Dosage on Clinical and Biochemical Parameters.

| **Outcome** | **Effect** | **F** | **p** | **Partial η^2^** |
| --- | --- | --- | --- | --- |
| Weight | Time | 31.56 | <0.001 | 0.135 |
|  | Time * Group | 2.45 | 0.551 | 0.006 |
|  | Time * Dosage | 2.51 | 0.435 | 0.003 |
| BMI | Time | 28.66 | <0.001 | 0.124 |
|  | Time * Group | 1.23 | 0.296 | 0.012 |
|  | Time * Dosage | 0.72 | 0.398 | 0.004 |
| WC | Time | 31.26 | <0.001 | 0.134 |
|  | Time * Group | 0.70 | 0.499 | 0.007 |
|  | Time * Dosage | 0.47 | 0.494 | 0.002 |
| SBP | Time | 10.98 | 0.001 | 0.052 |
|  | Time * Group | 0.59 | 0.555 | 0.006 |
|  | Time * Dosage | 0.12 | 0.732 | 0.001 |
| DBP | Time | 20.07 | <0.001 | 0.090 |
|  | Time * Group | 0.09 | 0.914 | 0.001 |
|  | Time * Dosage | 2.03 | 0.156 | 0.010 |
| FPG | Time | 25.92 | <0.001 | 0.114 |
|  | Time * Group | 0.07 | 0.929 | 0.001 |
|  | Time * Dosage | 2.25 | 0.135 | 0.011 |
| PPG | Time | 19.66 | <0.001 | 0.090 |
|  | Time * Group | 0.80 | 0.451 | 0.008 |
|  | Time * Dosage | 1.52 | 0.218 | 0.008 |
| HbA1C | Time | 22.84 | <0.001 | 0.150 |
|  | Time * Group | 2.08 | 0.129 | 0.031 |
|  | Time * Dosage | 4.89 | 0.029 | 0.037 |
| Total cholesterol | Time | 7.92 | 0.006 | 0.052 |
|  | Time * Group | 0.26 | 0.770 | 0.004 |
|  | Time * Dosage | 1.16 | 0.283 | 0.008 |
| Values represent F-statistics from repeated measures ANOVA. Significant effects are indicated at p<0.05.  Abbreviations: BMI, body mass index; WC, waist circumference; SBP, systolic blood pressure; DBP, diastolic blood pressure; FPG, fasting plasma glucose; PPG, postprandial glucose; HbA1c, glycated hemoglobin; η², partial eta-squared. | | | | |

**Suppl. Table 3.** Genotype Distribution and Allele Frequencies of Selected SNPs in the Study Population.

| **SNPs** | **N** | **Genotype (%)** | | | **Intergroup difference**  **(χ^2^, p)** | **Allele f** | **Allele dbSNP Global f** |
| --- | --- | --- | --- | --- | --- | --- | --- |
| rs13376631 | 202 | AA (88.6) | AG (10.9) | GG (0.5) | 1.45, p=0.835 | p(A)=0.94 | p(A)=0.86 |
| rs12943590 | 196 | AA (20.4) | AG (51.5) | GG (28.1) | 4.83, p=0.295 | p(G)=0.54 | p(G)=0.73 |
| rs12752688 | 195 | CC (79.0) | CT (20.5) | TT (0.5) | 1.22, p=0.875 | p(C)=0.89 | p(C)=0.87 |
| rs12208357 | 178 | CC (95.5) | CT (4.5) | - | 3.30, p=0.192 | p(C)=0.98 | p(C)=0.94 |
| rs10213440 | 193 | CC (6.7) | CT (31.6) | TT (61.7) | 4.92, p=0.295 | p(T)=0.77 | p(T)=0.82 |
| rs2289669 | 195 | AA (31.8) | AG (51.3) | GG (16.9) | 3.62, p=0.460 | p(G)=0.43 | p(G)=0.60 |
| rs3792269 | 180 | AA (81.7) | AG (16.7) | GG (1.7) | 1.24, p=0.872 | p(A)=0.90 | p(A)=0.85 |
| rs2076828 | 195 | CC (21.0) | CG (47.2) | GG (31.8) | 7.08, p=0.132 | p(C)=0.45 | p(C)=0.57 |
| rs11212617 | 194 | AA (23.2) | AC (47.9) | CC (28.9) | 9.51, p=0.050 | p(C)=0.53 | p(C)=0.44 |
| rs594709 | 202 | AA (42.6) | AG (45.0) | GG (12.4) | 1.25, p=0.869 | p(G)=0.35 | p(G)=0.37 |
| Differences between clinical groups were assessed using chi-square (χ²) tests. Allele frequencies are shown for the study sample (f) and compared with dbSNP global frequencies.  Abbreviations: SNP, single nucleotide polymorphism; dbSNP, Single Nucleotide Polymorphism Database. | | | | | | | |
